# Value Change, Value Conflict, and Policy Innovation: Understanding the Opposition to the Market-Based Economic Dispatch of Electricity Scheme in India Using the Multiple Streams Framework

**DOI:** 10.1007/s11948-022-00402-4

**Published:** 2022-11-14

**Authors:** Nihit Goyal, Kaveri Iychettira

**Affiliations:** 1 Faculty of Technology, Policy and Management, Delft University of Technology, Jaffalaan 5, 2628 BX Delft, Netherlands; 2 School of Public Policy, Indian Institute of Technology Delhi, New Delhi, India; 3 Belfer Center for Science and International Affairs, Harvard Kennedy School, Cambridge MA, United States of America

**Keywords:** Indian energy transition, Market-based economic dispatch of electricity (MBED), Multiple streams framework (MSF), Policy innovation, Policy process, Renewable energy, Value change, Value conflict

## Abstract

As policy innovation is essential for upscaling responsible innovation, understanding its relationship to value change(s) occurring or sought in sociotechnical systems is imperative. In this study, we ask: what are the different types of values in the policy process? And, how does value change influence policy innovation? We propose a disaggregation of values and value change based on a four-stream variant of the multiple streams framework (MSF), a conceptual lens increasingly used for explaining policy innovation in sociotechnical transitions. Specifically, we posit that the values that ‘govern’ problem framing, policy design, political decision making, and technological diffusion can evolve relatively independently, potentially leading to value conflict. We apply this framework to the ongoing case of the market-based economic dispatch of electricity (MBED) policy in the Indian energy transition using content analysis. We find that the MBED scheme—with its emphasis on efficiency (problem), economic principles (policy), low-cost dispatch (technology), and centralization (politics)—attempts value change in each stream. Each instance of value change is, however, widely contested, with the ensuing value conflicts resulting in significant opposition to this policy innovation. We conclude that a disaggregation of values based on the MSF can facilitate an analysis of value change and value conflict in sociotechnical transitions and lay the foundation for systematically studying the relationships among technological change, value change, and policy change.

## Introduction

The field of responsible research and innovation aims to “promote the inclusion of public values in innovation processes to address grand challenges” (Von Schomberg, 2013). As part of this, the value sensitive design approach, for example, is a “theoretically grounded, interactional approach to the design of technology that accounts for human values in a principled and comprehensive manner throughout the design process” (Umbrello, 2018, p. 239). Such values include, among others, environmental sustainability, equity, informed consent, and well-being (Friedman et al., 2013). Policy innovations in science & technology policy as well as the specific domain(s) of the innovation or technology are essential for the achievement of this objective (De Saille, 2015; Fitjar et al., 2019; Pacifico Silva et al., 2018).

Value change—induced by technological innovation or otherwise—influences the policy process and, thereby, policy innovations in the context of sustainability transitions, i.e., “a set of processes that lead to a fundamental shift in sociotechnical systems” (Markard et al., 2012, p. 956). Illustratively, it affects the discourse on the role of new technologies in society (Sousa & Marques, 2013), reduces the social acceptance of emerging technologies, such as sustainable heating systems, and constricts policy alternatives (De Wildt et al., 2021a), leads to an expansion of policy issues to new venues of governance (Feindt, 2012), increases political vulnerability of policy implementation and—consequently—sustainability transitions (Kay & Ackrill, 2012). It is, therefore, important to study the relationship between value change and policy innovation.

In this study, we address the following questions: what are the different types of values in the policy process? How does value change influence policy innovation? To do so, we conceptualize values in the policy process using a four-stream variant of the multiple streams framework (MSF). The literature on the ethics of science & technology has made a distinction between values in the engineering community, the policy making community, and the general society (Van De Poel et al., 2020). We build on this insight to propose a typology of values based on the MSF. Specifically, we posit that the values that influence problem framing, policy design, political decision making, and technological diffusion in sustainability transitions might be different from one another. As the streams evolve parallelly, so can the values therein, potentially leading to value conflicts that affect the coupling among the streams and reduce the likelihood of policy innovation.

We contend that the MSF is an appropriate and effective theoretical framework to address the research questions posed in this study. Originally formulated to explain agenda setting in the policy process, the MSF has subsequently been used to study policy adoption as well (Kingdon, 1995; Zahariadis, 1992). Moreover, research in science & technology studies has adapted the framework—through introduction of a technology stream—to study (policy making in) sociotechnical transitions (Elzen et al., 2011; Voß, 2007). In comparison to the advocacy coalition framework (Sabatier, 1988), which is more applicable to a stable policy subsystem rather than a nascent one (Jenkins-Smith et al., 2018), the MSF is especially useful under conditions of ambiguity (Zahariadis, 2003), often present in the form of fluid participation, long-term uncertainty, and rapid changes in dynamics during technological development and diffusion (Nathan et al., 2008). In addition, the MSF can also support a normative approach to the resolution of value conflicts for responsible innovation by shedding light on various dimensions of success (Goyal, 2021a) and emphasizing context-specific policy entrepreneurship in sustainability transitions (Goyal et al., 2020).

We map this conceptualization of values within the MSF onto the case of the proposed market-based economic dispatch of electricity (MBED) scheme in the ongoing Indian energy transition, from a carbon-intensive energy system to one prioritizing renewable energy. For our study, the MBED scheme represents a case of a policy proposal that is on the government agenda but is facing significant opposition during adoption, at least in part, due to the value conflict brought to the fore by the proposed policy design. In contrast to a typical MSF analysis—which prioritizes explaining the process of agenda setting or policy adoption—with this case study, we focus on understanding the opposition to policy innovation based on a disaggregation of values in the policy process. An investigation of this case: (i) demonstrates the applicability of our analytical framework for differentiating values and uncovering value change in the policy process; and (ii) indicates how value change can create value conflict that underpins resistance to a new policy. Further, this exercise is useful from a policy perspective as the Indian renewable energy transition is critical for global sustainability and will play a key role in achievement of the target to limit global temperature rise to 1.5 degrees Celsius by 2050. In addition, identifying key value conflicts in the Indian energy transition can facilitate lesson-drawing for the renewable energy integration in other low- and middle-income countries, which share several similarities with the policy context in India.

We make several contributions to the literature. First, we draw on the literature in social psychology, policy studies, and political science to offer a more granular disaggregation of societal values than presently found in studies on ethics of science & technology. Second, we contribute to the literature on value-sensitive design by systematically conceptualizing *and* operationalizing values in the context of our analytical framework and case study. Third, we demonstrate the use of the MSF for systematically conceptualizing the interactions among stakeholders involved in the policy process in the context of a sociotechnical transition (Nathan et al., 2008), which paves the way for better understanding the mechanisms of value change in the future (De Wildt et al., 2021b). Fourth, and relatedly, by advancing the conceptualization of values, value change, and value conflict in the policy process, we enable the application of value sensitive design to policy formulation (Taebi et al., 2014). Fifth, we advance the scholarship on the MSF by elaborating on the role of values within the framework and correcting the neglect of active opposition to policy change within its scholarship. Sixth, we contribute to energy studies through the application of the policy innovation lens to understand the energy transition (Goyal et al., 2022). Seventh, we add knowledge on a key case of energy transition in the Global South, which is underrepresented in the literature (Besant-Jones, 2006; Dubash & Morgan, 2013; Vanegas Cantarero, 2020), and also diversify the analysis of energy policy in India (Goyal, 2021b).

The article is structured as follows. In the next section, we develop the analytical framework for this study. Subsequently, we briefly introduce the background to the case, and elucidate its relevance to the research questions and the analytical framework. Thereafter, we describe the research methods and present the findings of the study. Finally, we discuss the implications of this research and conclude the article.

## Analytical Framework

The analytical framework developed in this section comprises the theoretical arguments which address the research questions: *what are different types of values in the policy process? and, how does value change influence policy innovation?*

### Values, Value Change, and Value Conflict

Friedman & Hendry (2019, p. 24) define values as “what is important to people in their lives”. Other scholars have defined values as “concepts or beliefs, about desirable end states or behaviors, that transcend specific situations, guide selection or evaluation of behavior and events, and are ordered by relative importance” (Schwartz & Bilsky, 1987, p. 551); or, as “lasting convictions or matters that people feel should be strived for in general and not just for themselves to be able to lead a good life or realize a good society” (Van De Poel & Royakkers, 2011, p. 72). Examples of values are dignity, justice, privacy, safety, sustainability, and well-being (Friedman & Hendry, 2019; Van De Poel, 2018). Generally speaking, values are distinguished from goals—which are desired, rather than desirable—and considered to be more generic than beliefs, which are propositions held as facts by human beings (Shuchman et al., 1962). Further, Dignum et al., (2016) have made a distinction between procedural values—pertaining to decision-making procedures and rules in a given context—and substantive values, pertaining to the object under consideration (such as a technology).

The literature on ethics in science & technology has recognized that societal values are not absolute and change over time. Broadly, value change may occur due to certain events, generational turnover, moral progress, societal change, or technological innovation (Kendal & Raymond, 2019; Melnyk, 2021; Van De Poel, 2018). In her investigation of value change in the context of the energy transition, Melnyk (2021), for example, describes value change as “a gradual process… that expands the social functions of values to new dimensions of moral concern.” To enable a systematic study of value change, Van De Poel (2018) has developed the following taxonomy of value change: (i) emergence of new values; (ii) changes in relevance of values; (iii) changes in prioritization of values; (iv) changes in conceptualization of values; (v) changes in specification of values through norms and design requirements. Illustratively, De Wildt et al., (2021b) use probabilistic topic modelling—an unsupervised machine learning technique—to classify long-term value change in energy technologies based on this taxonomy.

Scholars have posited that value change can result in value conflict or value tension. For example, in their study on the effect of digitization on the energy system, Niet et al., (2021) find that emerging values—such as autonomy, balance of power, control over technology, and equity—conflict with ‘anchored’ or ‘institutionalized’ values, such as affordability, (cyber)security, privacy, reliability, and sustainability. They identify three specific value tensions that result from this conflict: level playing field, preserving system functionality, and stimulating self-determination. Further, Dignum et al. (2016) have distinguished conflict between values (i.e., inter-value conflict) and conflict in the operationalization of the same value (i.e., intra-value conflict) in their analysis of the public debate on shale gas in the Netherlands. However, an understanding of value conflicts based on the source(s) of value change is presently missing in the literature.

We assert that to better understand value conflict, it is important to disaggregate the source(s) of value change in a sociotechnical system. While numerous scholars only differentiate values pertaining to technology generally from values in society, Van De Poel et al. (2020) add a further distinction between values in the engineering community, the policy making community, and the general society. We build on this insight to propose a typology of values, classify the sources of value change, and identify the types of value conflicts in sociotechnical transitions using the MSF.

### The Multiple Streams Framework

The MSF originally explained agenda setting using five key elements: three streams, windows of opportunity, and policy entrepreneurship (Kingdon, 1995). The problem stream represents elite perception of societal conditions based on focusing events, indicators, and feedback from previous policy implementation. The policy stream depicts the ‘mutation’ and ‘recombination’ of policy alternatives based on selection criteria such as technical feasibility, financial viability, and value acceptability. The politics stream, meanwhile, encapsulates characteristics such as balance of ‘interest’, party ideologies, public mood, and electoral activity. A key premise of the framework is that the streams are relatively independent and only ‘loosely coupled’. Kingdon argued that an issue is placed on the policy agenda when the streams are aligned, or fully coupled, through policy entrepreneurship. Entrepreneurial activities are more likely to succeed in coupling the streams during windows of opportunity, which are time periods when the government has higher expectation or willingness for undertaking policy action.

Scholars have since applied the MSF to analyze policy making in over 300 cases, at various levels of government in diverse policy areas around the world (Jones et al., 2016). The framework has been found to be especially insightful under conditions of ambiguity—in the form of problematic preferences at the societal level, unclear ‘technology’ linking the problem and the solution, and fluid participation in decision making (Cohen et al., 1972)—which is increasingly a feature of complex sociotechnical systems (Zahariadis, 2003, 2008). Further, though the framework was initially conceived to explain agenda setting, it has subsequently been extended to investigate policy adoption (Goyal & Howlett, 2020a; Herweg et al., 2018; Howlett et al., 2015; Zahariadis, 1992). Moreover, studies have demonstrated the applicability of the framework outside the Global North, including in countries in Asia and Latin America (Goyal, 2021c; Sanjurjo, 2020; Van Den Dool, 2022), while also incorporating the role of policy entrepreneurship in fostering policy innovation (Goyal et al., 2020).

Although the role of technologies in the policy process was largely implicit in the original framework, subsequent research has bridged this gap. As an acknowledgement of the co-evolutionary dynamic between policy making and technological innovation (Hoppmann et al., 2014; Schmidt & Sewerin, 2017), scholars have adapted the MSF by introducing a technology stream (Elzen et al., 2011; Voß, 2007). This stream captures events and activities that influence technological development, such as research, patenting and licensing, business venture creation, and technology diffusion and transfer (Goyal, 2019; Goyal et al., 2020). Supported by technology constituencies (Goyal & Howlett, 2018)—comprising technologists, manufacturers, suppliers, service providers, users, and other stakeholders who share an interest in the diffusion of specific technologies—the evolution of this stream might shape not only technological trajectories but also the policy making streams (Goyal & Howlett, 2020b). Illustratively, Goyal et al., (2021) hypothesize the relationship of the technology stream to problem, policy, and politics and apply this four-stream variant of the MSF to explain the emergence of the General Data Protection Regulation (GDPR) in the European Union (EU).

However, research on the MSF has not systematically conceptualized the role of values—and, thereby, value change or value conflict—in policy making. This is not to say that the topic has been neglected in this literature. In fact, values play an important part in issue framing in the problem stream (Knaggård, 2016) and value acceptability is a key criterion determining the ‘survival’ of policy ideas in the policy stream (Kingdon, 1995). Further, research in the area of morality policy has studied policy processes where policy change to effect value change receives explicit focus (Mourão Permoser, 2019; Sharma, 2008). Yet, value acceptability is simplistically conceived as conformity with the values of members of the policy community, i.e., “mainly a loose connection of civil servants, interest groups, academics, researchers and consultants (the so-called hidden participants), who engage in working out alternatives to the policy problems of a specific policy field” (Herweg, 2016, p. 132). As a result, an account of how values and value change influence the streams in the MSF is currently missing.

In the next subsection, we conceptualize values within the MSF to lay the foundation for our empirical analysis.

### Conceptualizing Values Within the Multiple Streams Framework

As mentioned previously, the problem stream represents elite perceptions of societal conditions based on indicators, focusing events, and policy feedback. The values of elites, and the general society, can shape these perceptions and influence problem framing (Knaggård, 2015). Illustratively, the gradual rise in prominence of the value of sustainability—especially since the work of World Commission on Environment and Development (1987)—has contributed to the framing of climate change as a problem (Melnyk, 2021). Further, values can affect—and in turn be affected by—the indicators that are created and used to monitor societal conditions. The OECD, for example, has proposed an alternate measurement of well-being (rather than GDP) and created the Better Life Index to prioritize the value of ‘well-being’ over ‘economic growth’ (Bache & Reardon, 2013).

Value change in this stream may occur due to changes in societal context, generational turnover, or changes in elite composition. Abramson et al. (1997), for instance, have noted a change from economic, materialist, and physical values to values of autonomy and self-expression—a phenomenon they term as postmaterialism—in societies that witness steady economic development. In addition, focusing events can also contribute to value change. Illustratively, the aftermath of the Fukushima accident prompted a prioritization of safety as a value in Germany (Wittneben, 2012). Similarly, the 2008 financial crisis—arguably itself associated with a change in values of elites towards profit maximization—contributed to a change in the values surrounding macroeconomic regulation from efficiency to prudence (Baker, 2013) and the COVID-19 pandemic lead to prioritization of security, stability, and threat avoidance (Daniel et al., 2021; Steinert, 2021).

In the policy stream, policy communities influence the evolution of policy alternatives based on selection criteria such as budgetary workability, technical feasibility, and value acceptability (Kingdon, 1995). How these criteria are prioritized and which values inform acceptability can differ from one policy community to another. For example, while economic efficiency might be an acceptable or even a necessary value in the case of a public budgeting reform, the value of secrecy is likely to be paramount in the case of a national security initiative (McConnell, 2010). Further, values may affect the types of policy instruments selected, such as command-and-control versus incentive maximizing, or even whether policy action is deemed appropriate in a specific situation.

Values in the policy stream can change due to the evolution of ideas pertaining to public policy or the emergence of new policy communities. The shift from Keynesianism to monetarism, for example, was a change in the value of government intervention in the economy that was influenced, at least in part, by prevailing macroeconomic theories of the time (Hall, 1993). Recently, the advocacy for ‘libertarian paternalism’ (Thaler & Sunstein, 2009)—and the ‘behavioral turn’ in public policy—can also be viewed as an ongoing change in the policy stream that aims to reconcile the seemingly conflicting values of freedom of choice and government intervention to influence welfare. Further, values beyond economic efficiency—illustratively, the value of public participation in policy design (Roberts, 2004) or the values of policy durability, resilience, or robustness in longterm decision making (Regan et al., 2005; Turner, 2020)—have also gained traction in some policy communities.

The technology stream depicts research and development activities by scientists, engineers, and businesses to create and diffuse new technologies in the sociotechnical system (Goyal, 2019; Goyal et al., 2020). The values and ethical positions of these actors influence the values in the technology stream (Jin & Drozdenko, 2010; Ustek-Spilda et al., 2019). In addition, a technology system may impose values or have values ascribed to it (Shilton et al., 2013; Tang et al., 2020). Therefore, the values in this stream are context dependent and vary from one application area or domain to another. For instance, privacy might be an salient value in the case of information and communication technologies (Xu et al., 2012) while safety might be a key consideration for energy infrastructure (Wittneben, 2012).

Values in the technology stream may change because of the turnover in the actors involved in this stream or the processes of technological development and diffusion. Such value changes may be intentional or unintentional. The field of value sensitive design, for example, aims to embed normative considerations into technology from the beginning of the design process (Friedman et al., 2006). In other words, it strives to promote the value of ‘responsibility’ as complementary to innovation or novelty in the technology stream.

Finally, the politics stream models characteristics such as the public mood, party ideologies, and interest group activities. Political scientists have made a distinction between private values and political values of citizens (Zhai, 2021). Strenze (2021), for example, argued that while political values have become more postmaterialistic in the Unites States and Western Europe since 1970s, values related to work have become more materialistic. At the outset, values such as authoritarianism, emancipation, and democracy shape domestic politics and, thereby, the development of this stream. Illustratively, Welzel (2021) has argued that democratic backsliding is not a global phenomenon and is occurring only in cultures where emancipative values remain under-developed. Further, party ideologies themselves represent several values that influence citizens’ support to political candidates. Also, interest group activities are influenced by values that affect state-society and state-business relationship, such as corporatism or pluralism (McFarland, 2007; McLennan, 1989; Truman, 1951).

Values in the politics stream may change due to generational turnover, national context, or electoral activity. In a comparison of citizenship norms among adolescents in 21 liberal democracies, for example, Hooghe and Oser (2015) found a shift from the value of duty to the value of engagement. Kostelka and Blais (2021) have ascribed a decline in the value of electoral participation in post-war democracies to generational turnover and a rise in the number of elective institutions. In the case of European societies, Savelyev (2016) has argued that the national context is likely to have played a more significant role in value change rather than population turnover. Further, Mccann (1997) has found that, in the United States presidential election in 1992, choosing sides in the election itself led to value change among the voters.

Illustrative values that can influence the evolution of each stream and the potential sources of value change within the stream are summarized in Table 1. The above discussion underscores that (i) values influence each stream of the MSF, and (ii) value change can occur in any stream of the MSF. Following the logic of the MSF, value change might increase the likelihood of policy innovation when it enhances the alignment among the streams (Elzen et al., 2011). However, value change might create a value conflict *within* a stream, for example, leading to contestation in problem framing (De Wildt et al., 2019), policy design specification (Haelg et al., 2020), level playing field for technology (Niet et al., 2021), the emergence of new political parties (Ford & Goodwin, 2014; Marthaler, 2008), adversarial political practices (Engels, 2008; Nevitte, 2000; Wang & You, 2016), or competing coalitions (Meijerink, 2005). Alternatively, value change might create conflict *between* the streams, for example, if the values in the technology stream seem irreconcilable with the values in the policy stream (Kernaghan, 2014) or the values in the policy stream seem irreconcilable with the values in the politics stream (Laes & Bombaerts, 2022). As value conflict within a stream can prevent ripening of the stream and value conflict between streams can prevent coupling, we posit that value conflict within or between the streams can be a source of opposition to policy innovation and, thereby, affect the pace of a sociotechnical transition.

**Table 1:** Values and value change in the multiple streams framework

Stream	Illustrative values	Potential source of value change
Problem	Autonomy; economic growth; safety; security; sustainability; well-being	Elite composition; generational turnover; societal development
Policy	Economic efficiency; freedom of choice; policy durability; public participation; secrecy	Changes in policy communities; ideational change
Technology	Innovation; privacy; responsibility; safety	Changes in technology constituencies; technology diffusion
Politics	Corporatism; duty; emancipation; engagement; equality	Electoral activity; generational turnover; societal context

## The Case of the Market Based Economic Dispatch of Electricity Policy in India: Background and Case Relevance

In this section, we first present a background to help contextualize the MBED scheme: the Indian energy transition, with a particular focus on the issues relating to the integration of renewable energy. We then briefly introduce the case itself, describe its relationship to the analytical framework presented above, and explain its contribution to addressing the research questions.

### Background: The Historical Context of the Indian Energy Transition

In the 1990s, when electricity sector reforms began sweeping high-income countries, the electricity sector in India was organized in a vertically integrated, state-owned fashion, while also operating under chronic shortage of electricity generation capacity. In an attempt to attract private capital into the sector, increase efficiency, and increase costrecovery for electricity distribution companies, the Electricity Act of 2003 was passed by the Indian Parliament as a partial attempt at liberalizing the sector. Under this Act, although the union government exercises authority over electricity generation and inter-state transmission, the state governments exercise authority over generation, intra-state transmission, and distribution. The sector continues to be ridden with issues such as unreliable access, financially unviable state electricity utilities, and technical and commercial inefficiency in the distribution segment (Dubash et al., 2018; Haldea, 2001).

The growth of renewable energy in India has occurred in this environment. In 2008, the then Prime Minister Manmohan Singh announced a National Action Plan on Climate Change, which included an ambitious target for promoting solar energy through a “National Solar Mission” (Rastogi, 2011). This target spawned much private sector investment and public discussion on a roadmap for achieving the goal. In November 2013, Niti Aayog, the think tank of the Government of India initiated a “stakeholder driven analysis of the opportunities and barriers to rapid deployment of renewable electricity” (Niti Aayog, 2015).

In 2014, when a new government was elected, Prime Minister Narendra Modi adopted a stronger push on renewable energy, which was seen as a solution to both India’s energy demand and sustainability imperatives. Encouraged by dramatically declining prices of solar and wind energy, the Prime Minister announced a several-fold increase in targeted renewable energy capacity, with a new target of 175 GW by 2022. In contrast, the installed capacity, as of February 2022, was 91 GW (Government of India, 2022). In 2019, India announced an even more ambitious target of 450 GW of renewable energy by 2030, signaling a continued commitment to renewable energy.

Unlike conventional sources of electricity generation, such as coal, gas, or hydro power, solar energy and wind energy are intermittent. Intermittency is a term used to capture two characteristics: firstly, variability in the generation from solar and wind over time; and, secondly, uncertainty associated with this variability (Verzijlbergh et al., 2017). A widely accepted way of dealing with this intermittency is through the concept of flexibility, defined by the International Energy Agency (2011) as “the extent to which a power system can modify electricity production or consumption in response to variability, expected or otherwise.”

One way to increase flexibility is through better use of the electricity transmission network with appropriate institutional mechanisms, which are crucial for coordinating this system of points of generation and demand across space. This coordination becomes more important in the presence of renewable energy, because weather patterns are uncorrelated over large distances (Iychettira, 2021; Verzijlbergh et al., 2017). Markets, or similar economic dispatch, built close to real time are a crucial way of organizing this coordination, especially for integrating higher shares of intermittent renewable energy in the power system (Neuhoff et al., 2013). However, implementing such markets, or economic dispatch, requires policy innovation.

### Case Selection and its Relationship to the Analytical Framework

The case analyzed in this article is the proposal for a new design for the operation of the day-ahead market in India, known as the MBED scheme. The proposal for the MBED scheme was put forth by the Staff of the Central Electricity Regulatory Commission (CERC) in 2018 (CERC, 2018b), along with other market redesign proposals such as the real-time market (CERC, 2018a). The justification for the MBED proposal was the following: when implemented, the scheme would increase efficiency in scheduling and dispatch, reduce the cost of electricity supply, and enhance flexibility of the power system to integrate intermittent renewable energy sources. Thus, the proposal sought to foster value changes in the Indian energy transition. However, the scheme was strongly opposed by actors across the board, including state electricity utilities, policy research institutes, independent analysts, electricity plant developers, and politicians.

We select this case, because it is situated well along the various dimensions relevant to the theoretical argument of this paper; dimensions such as problem framing, policy design, political decision making, and technological diffusion, all of which can evolve relatively independently, potentially leading to value conflict. The analytical framework developed earlier indicates that value change can lead to value conflict within a stream or between the streams, which could then constrain policy innovation. Thus, the objective of the case is to: (i) test the applicability of the above conceptualization of values using the MSF; and (ii) uncover the key value conflicts, if any, that constrain the adoption of the proposed MBED policy. We employ the case study to answer the following empirical questions: what are the key values and value changes in the proposed MBED scheme in India? How do value conflicts contribute to the opposition to the MBED scheme in the context of the increasing share of intermittent renewable electricity in India? A closer look at the case shows that a key reason for this opposition is the value changes embodied in the policy proposal and the value conflicts engendered by it. As a result, while the other real-time market had come into operation by June 2020, the proposal for the MBED scheme did not move beyond the policy formulation stage, and remains un-implemented even four years later, as of this writing.

## Research Methods

In this section we present the data sources and describe the analysis techniques used in the study.

### Data Collection

We base our analysis on documents from government websites, interviews, news articles, policy reports by think tanks, and secondary literature. While we refer to much background material to understand the context of the policy proposal, our primary data sources for value conflicts are the discussion paper on the MBED scheme released by the Staff of the CERC, along with the 37 official responses to it as part of the public consultation process. The policy proposal and the responses received are publicly available (CERC, 2018b). We triangulate this data with information from conversations and interviews conducted with various stakeholders from 2018 to 2020. We conducted more than 25 interviews across more than three states in India with officials involved in the electricity sector, working for organizations such as distribution companies, regulatory commissions, power procurement agencies, and departments involved in the trading of power. These organizations were selected because their operations and financial outcomes would be most affected by the proposed policy, if implemented. The interviews were conducted in a semi-structured manner, with questions focusing on capabilities, perceptions, and preferences related to the implementation of and outcomes from market-based trading mechanisms.

### Analysis Techniques

We examine this data using content analysis with pre-defined coding (Krippendorff, 2018). Specifically, we code events, activities, and viewpoints mentioned in the text into the four streams: problem, policy, politics, and technology. Illustratively, statements describing an issue—such as an undesirable level of an indicator—were coded as part of the problem stream, while those mentioning the design characteristics of the scheme were coded as part of the policy stream. Similarly, discussion on the likely effect of the policy on technology and infrastructure were captured under the technology stream and the relationship of the policy with the distribution of administrative or political authority—for example, through centralization or decentralization—and government decision making—for example, through electoral prospects, interest groups, or party ideologies—were labelled as part of the politics stream.

Subsequently, we scanned the coded data for implicit or explicit values by referring to the characteristics defined earlier: (i) concepts or beliefs; (ii) referring directly or indirectly to the desirable; (iii) described with little or no reference to a specific context; and (iv) employed for prioritization of alternatives. Illustratively, consider the statement: “it [self-scheduling] leaves several low-cost generation capacities partially or sub-optimally utilized” (CERC, 2018, p. 9). This viewpoint is coded as part of the problem stream as it pertains to an issue caused by feedback due to prior policy implementation. Further, it is also coded as denoting the value ‘efficiency’, as a desirable characteristic of the electricity system that is presumably applicable under almost all circumstances and can help in optimum utilization of electricity generation capacity. Finally, we compared values embedded in the MBED proposal against those in the comments to the proposal to identify synergies and conflict.

## Results

In this section we present the findings of our analysis regarding the value changes explicitly or implicitly embodied in the MBED scheme and the value conflicts that drive, to a significant extent, the opposition to this scheme.

### Value Change and the Market-Based Economic Dispatch of Electricity Policy

The idea of designing the electricity market to facilitate renewable energy integration is dominant in policy communities across Europe and the United States (Neuhoff et al., 2013; Sensfuss et al., 2008). While it is difficult to state with certainty exactly when this idea was ‘transferred’ to India, it found a mention in a report released by the Niti Aayog (2015) that had been co-authored by the Regulatory Assistance Project, a US-based think tank. The idea of addressing the ‘problem’ of renewable energy integration through market design was re-iterated through ‘Greening the Grid’, a joint project between the United States Agency for International Development (USAID) and the Ministry of Power, Government of India (NREL & POSOCO, 2017). Other actors advocating for this approach included academics (Das et al., 2020; Singh, 2010) and national and international think tanks (IEA International Energy Agency, 2021; Spencer et al., 2020).

In this ‘primeval soup’ of policy ideas, to use Kindgon’s phrase for the policy stream (Kingdon, 1995), one actor exercised policy entrepreneurship: a career-bureaucrat, holding the position of Joint Chief (Regulatory Affairs) at the Central Electricity Regulatory Commission (CERC). Since about 2015, they liaised with academia, the bureaucracy, policy research institutes, and power sector professionals across the country through meetings, reports, white papers, and workshops to discuss views concerning electricity market design with the objectives of enhancing efficiency, reducing cost, and increasing renewable energy penetration, thereby ‘softening’ the policy idea of the MBED scheme.

A window of opportunity opened possibly in 2018, when after several decades India was expected to become electricity ‘surplus’ (The Economic Times, 2018), and when strong targets were set for investment in renewable generation capacity. The electricity system in India had long struggled to cope with rapidly rising electricity demand due to its population growth, improving standard of living, and increasing urbanization. As a result, electricity requirement invariably exceeded supply, resulting in a persistent power deficit. Through a concerted effort at augmenting electricity generation capacity, along with a less-than-expected increase in demand, gradually the situation improved (CEA, 2008, 2017). The availability of ‘excess’ electricity in the grid, in combination with a continued strengthening of policy support for renewable energy, created an opportunity for re-thinking scheduling and dispatch of electricity in the country. Later that year, the Staff of the CERC proposed a fundamental re-design of the electricity market in India, including through the introduction of the MBED scheme for the day-ahead market (CERC, 2018).

In its proposal for the MBED scheme, the Staff of the CERC framed purchasing of nearly 90% electricity through ‘self-scheduling’ by distribution utilities as the overarching problem. This practice, it argued, hides the ‘visibility’ of alternative sources of electricity and, consequently, creates inefficiency in electricity procurement. The discussion paper, for example, contended: “it [self-scheduling] leaves several low-cost generation capacities partially or sub-optimally utilized. This is because the DISCOMs do not have visibility of other cheaper options nor do they have the right to requisition/schedule power from the generating stations with which they do not have a contract” (CERC, 2018, p. 9). Further, the Staff opined that self-scheduling “often constrains optimum utilization of renewable sources of energy” and leads to curtailment (CERC, 2018, p. 14). Thus, the Staff espoused efficiency as a key value, and transparency and renewable energy integration as other values in the problem stream.

To address the problem, the Staff proposed the market-based economic dispatch of electricity as the policy solution. The idea was to significantly, if not completely, shift electricity procurement from the prevailing approach of self-scheduling through long-term contracting to short-term electricity purchase on a national spot market. The paper discussed a detailed design for the scheme and presented a pathway for transitioning from the current system to this more efficient one. In doing so, the Staff sought to emphasize optimization as the sole value for the day-to-day operation of the sector. This was stated explicitly in the discussion paper: “This model would function on a day-ahead time horizon and schedule and dispatch all generation purely on economic principles, subject of course to technical constraints” (CERC, 2018, p. 1).

This proposal also sought to accelerate value change in the technology stream. Despite the aggressive push on renewable energy, a complex policy ‘mix’ mediates capacity addition and operation in the electricity sector in India. In contrast, the MBED scheme would incentivize the operation of electricity generation plants with the least marginal cost, lead to the creation of a larger balancing area, and signal the need for investment in ‘flexible’ sources of generation (Newbery et al., 2018). As a result, the scheme would prioritize low (variable) cost and decarbonization as the values in the technology stream. The discussion paper, for example, highlighted: “Those generators whose variable cost are above the MCP [market clearing price], would not be dispatched but will recover their fixed cost through existing contracts” (CERC, 2018, p. 54).

Finally, the MBED proposal envisioned a significant change in the politics of the Indian energy transition as well. In the discussion paper, the Staff stated: “The existing arrangement of self-scheduling of the long-term contracts described above should ideally hold good during the transition period (of say one year), after which all such generators as well as the DISCOMs with whom they have contracts should also be mandated to participate in the day ahead Market Based Economic Dispatch system” (CERC, 2018, p. 38). Embedded in this design is the notion of centralization of policy making in electricity generation and dispatch as a necessity for enhancing their operational efficiency.

In 2021, three years after the CERC proposed the MBED scheme, the Ministry of Power, Government of India, circulated another discussion paper that acknowledged the challenges in the adoption of the scheme (Ministry of Power, 2021a). Later that year, a press release announced that the MBED scheme would be implemented in phases, starting with a “consensual and phased approach” in April 2022, where only interstate generating stations would participate (Ministry of Power, 2021b). Yet, even as of this writing, the MBED scheme is yet to be adopted. One challenge widely acknowledged, including in the discussion paper by the Ministry of Power, is the lack of adequate working capital in state electricity distribution utilities, which prevents them from engaging in short-term market transactions of the type proposed in the MBED scheme.

A closer look into the perceptions and preferences of a wider variety of actors involved in the public consultation process, however, reveals deeper value conflicts that go beyond seemingly financial constraints, such as the working capital. In particular, the proposed Phase 1 of the MBED scheme works around some of the key value conflicts—such as centralization versus federalism—by mandatorily including only those generating stations that are *a priori* contractually obligated to participate in transactions across state borders. The press release states: “Ministry of Power noticed substantial alignment amongst all key stakeholders on… the process to be followed for implementing Phase 1 of MBED starting with the mandatory participation of the Inter State Generating Stations” (Ministry of Power, 2021b).

Thus, value conflicts are a key source of opposition to the proposed MBED scheme. In the following sub-section, we uncover the different value conflicts at play and how they shape the opposition to the MBED proposal.

### Value Conflicts in the Market-Based Economic Dispatch of Electricity Scheme

The comments received by the Staff of the Commission clarified the values enshrined in the MBED scheme and also expressed opposition to many of them. First, commentators challenged the problem frame adopted in the proposal for the MBED scheme and presented a more diverse account of the values in the problem stream. The notion that the ‘problem’ of self-scheduling was caused by the lack of visibility of electricity generation alternatives was contested. One commentator, for example, offered a different interpretation of inefficiency in electricity dispatch, in the process presenting a possibly competing value of statehood: “…some state DISCOMs do not want to dispatch cheapest cost power, that they would rather dispatch in-state power and keep the money within their system than send it to out- of-state generators” (Chandra, 2019, p. 2). Meanwhile, a state utility questioned efficiency as the principal value in the problem stream: “…the State Governments have developed power projects at different locations in geographical regions keeping in view the regional power balance and also keeping in view region specific growth perspectives and such project development was not with purely commercial view” (MSEDCL, 2019, p. 1). Other values that are invoked in response to the discussion paper include uninterrupted electricity supply (TPTCL, 2019) and consumer welfare (KISPL, 2019).

Second, responses to the MBED proposal demonstrated conflict in values in both the process and substance of the policy design. Regarding the process, commentators questioned the merit in a ‘big bang’ reform and made a case for an evolutionary approach to market design (Chandra, 2019; POSOCO, 2019; TERI, 2019). Illustratively, a state distribution utility recommended implementation in a single state on a pilot basis “to evolve the methodology in a better way prior to full-fledged nation-wide implementation” (MSEDCL, 2019, p. 4). Meanwhile, a regulator in Bihar hinted at the persistence of norms in energy governance, referring to long-term contracting as “the only way we know how to procure electricity.” These critiques of the proposal highlight a value conflict between comprehensive rationality and incrementalism as an approach to decision making. While the notion of comprehensive rationality is based on clarifying ends (i.e., objectives) and then identifying means to achieve them through exhaustive analysis, the idea of incrementalism considers the selection of ends and means as closely intertwined, and step by step progress based on stakeholder agreement as the appropriate way of improving the system (see Lindblom, 1959). In addition, a respondent called for a consultative process of policy formulation—especially considering the wide implications of the reform—rather than reliance only on modelling and simulation (L&T Financial Services, 2019, p. 1). Further, the Staff was also urged to value transparency in modelling and analysis: “all data used in models and simulations… should be made publicly available… Public consultation cannot be done on the back of private analysis” (Chandra, 2019, p. 10). These comments pitted the value of technocracy embodied in methods such as modelling and simulation used to legitimize policy design, as against the values of transparency, openness and social representation in the process of designing the policy.

Questions were raised about the design of the MBED scheme as well. Distribution utilities, for instance, valued certainty and were wary of the risk and uncertainty associated with short-term contracting. A state-level energy bureaucrat in the state of Madhya Pradesh (personal communication, May 2018) speculated about the fallout of the scheme: “without a contract, what if the generator sold their power to a different entity?” In another instance, the regulator in the state of Karnataka (personal communication, July 2018) compared dealing with price volatility in the electricity market as akin to gambling. Further, the principle underlying the distribution of efficiency gain that might ensue from implementation of the policy was heavily contested. While the Staff had proposed a 50:50 split between the electricity generating company and the distribution utility to which it was contractually bound, some contended that the latter should receive the entire profit as it, in effect, owned the electricity under the long-term contract (PKCL, 2019; Tata Power, 2019). More fundamentally, a respondent dismissed the idea of economic principles as the top value for policy-making: “…market is not the panacea for all these problems… a peremptory push for implementing new market mechanisms without considering the long reaching effects can further complicate the situation” (Haldia Energy Limited, 2019, p. 1).

Third, the proposed design of the MBED scheme conflicted with some values in the technology stream too. Specifically, the values of ‘low-cost’, and ‘better flexibility’ would take on a new importance in the technology stream with the introduction of the MBED scheme. Although the MBED scheme is designed to incentivize low-cost operation, stakeholders who would stand to lose (as compared to the status quo) raised concerns about uneven impacts on different power plants, and called for the value of a ‘level playing field’ to be upheld, thereby implying that all power plants should be treated the same. For instance, stakeholders raised concerns about the various ways in which the design might favor power plants with lower costs due to fuel linkages or access to concessionary coal, or those with an existing long-term contract and lower incremental cost of generation (IEX, 2019; PTC, 2019; TPTCL, 2019).

Fourth, the key value conflict within the politics stream concerns the conflict between the values of federalism and centralization in energy governance in India. In proposing a national merit order of electricity dispatch, the MBED scheme would promote the centralization of electricity governance in India (Adani Power, 2019). Numerous commentators also highlighted the illegality of the proposed scheme (APP, 2019; Tata Power, 2019; World Bank, 2019). For instance, one respondent bluntly observed: “The powers of regulating their [distribution utilities’] electricity purchase is also fully under the jurisdiction of the State Commissions… The discussion paper appears to propose infringement of powers of the State Commissions by the Central Commission” (CESC, 2019, p. 2). Another respondent contended that this centralization in decision making was at odds with the decentralized and diversified character of the energy system: “Power market is characterized by sudden change in parameters which require decision making power to be de-centralized for a better management and operation… a shift to centralized planning doesn’t hold merit” (IEX, 2019, pp. 8–9). Relatedly, and finally, the proposal was seen as conflicting with a procedural value in the politics stream: consensus building for decision making (Chandra, 2019).

The key value changes in the MBED scheme and the value conflicts are summarized in Table 2. The findings show that the value changes embedded in the MBED policy have created value conflicts not only within the problem stream and the policy stream, but—through them – between policy-technology and policy-politics. These explain, at least in part, the strong opposition to the policy and prevent coupling among the streams.

**Table 2:** Value changes and value conflicts in the proposed market based economic dispatch of electricity scheme in India

Stream	Value changes	Value conflicts
Problem	Economic efficiency Transparency Renewable energy integration	Efficiency versus autonomy Commercial operation versus regional development
Policy	Optimization	Comprehensive rationality versus incrementalism Technocratic approach versus public consultation Risk versus certainty (Equitable) Distribution of surplus Efficiency as (not) the guiding principle of policy design
Technology	Low cost of operation Flexibility	Low cost of operation versus level playing field Efficiency versus legacy
Politics	Centralization	Centralization versus federalism Efficiency versus constitutionality Top-down decision making versus consensus building

## Discussion and Conclusion

As policy innovation influences, and is influenced by, public values in (technological) innovation processes, it is important to examine its relationship to value change and value conflict. In this study, we addressed this gap by posing the following questions: what are the different types of values in the policy process? How does value change influence policy innovation? We conceptualized values, value change, and value conflict using a four-stream variant of the MSF, positing that problem framing, policy design, technological innovation, and political decision making are influenced by different—albeit potentially overlapping—values, which can evolve relatively independently. Our application of this analytical framework to the ongoing case of the MBED policy in India showed that the policy espoused the value of efficiency in the problem stream and the corresponding value of ‘economic principles’ in the policy stream. A consequence of its design was the prioritization of (variable) cost as the key value in the technology stream and the centralization of decision making in the politics stream. These value changes embedded in the MBED proposal created several value conflicts.

In the problem stream, respondents highlighted autonomy, economic growth, and regional development as values that influence issue framing. Meanwhile, in the policy stream, value conflicts involved both the process as well as the substance of policy design. In the process, incrementalism, consultation, and transparency were emphasized as values not reflected in policy formulation. Simultaneously, certainty and equitable distribution were articulated as values missing in the design, and the notion of economic efficiency as the sole value in the policy stream was contested. Further, the idea of a level playing field (that all technologies should be treated equally) came through as a value in the technology stream that could be threatened by the value of ‘low-cost operation’ espoused in the proposed design of the MBED scheme. At the policy-politics interface, the operationalization of economic principles in the proposal conflicted with the values of decentralization and federalism. The process of decision making was also seen as antithetical to consensus building. These value conflicts prevent coupling among the streams and result in opposition to the policy innovation.

The literature on ethics in science & technology has proposed a rather coarse disaggregation of values in terms of societal values and technological values. In a more fine-grained conception, Van De Poel et al., (2020) have distinguished the values in the engineering community from those in the policy making community and the general society. This study shows the analytical value of further differentiating (private) values in the general society from political values, and linking them to the streams of the MSF. The case of the MBED policy showed that the values influencing the problem stream, the policy stream, the technology stream, and the politics stream—albeit related—are distinct from one another. Further, values, value changes, or value conflicts can pertain to the process or the substance (Dignum et al., 2016) of each stream, as seen in the case of the policy stream and the politics stream in the MBED policy process.

Our approach provides a way for analyzing interactions among stakeholders in sociotechnical transitions over a long time frame. While the dynamics in our case have unfolded over a relative short timeframe, the MSF itself has been used to analyze policy processes lasting over a decade. Further, it has previously been proposed to study normative contestation (Elzen et al., 2011), learning (Goyal & Howlett, 2020b), and policy-technology co-evolution (Goyal et al., 2021) in transition processes. Also, the MSF has been operationalized in the context of multilevel governance (Goyal, 2021c; Rietig, 2021) as well as public participation in energy transitions (Goyal & Howlett, 2021). Therefore, its use can shed light on value changes and value conflicts in (policy processes in) sociotechnical transitions and create knowledge on the mechanisms of value change in these processes.

In addition, we answer the call of Taebi et al. (2014) for bridging the gap between research on the ethics of science & technology and policy studies by advancing the conceptualization of values, value change, and value conflict in the policy process. The resulting analytical framework extends the study of responsible innovation to the policy context. First, it can be used to empirically examine whether, to what extent, and whose values are included in the process of technological development and diffusion in sociotechnical transitions. Second, it paves the way for the application of approaches in responsible innovation, such as value sensitive design, to policy formulation.

This study also contributes to the scholarship on the MSF. Although value acceptability is a key criterion for policy advancement as per the framework, when and why are values across the streams (in)compatible, why and how do values change, and how do value changes influence policy innovation are questions that have not been studied adequately using the MSF. Here, we take a step in that direction. Further, existing research on the MSF is biased towards successful policy change and policy entrepreneurship. In one example of an exception, Llamosas et al. (2018) have deployed the MSF to argue that intragovernmental politics—aided by vested interests—in the energy system in Paraguay has resulted in policy stasis over a prolonged time period. Our analysis indicates that value conflicts can be another source of opposition or ‘resistance’ to policy innovation, and merit further attention by scholars of the policy process. Such research can foster further development of the MSF and enhance its utility for examining sustainability transitions.

Finally, our study has an important policy implication. The analysis of the policy process of the MBED scheme shows that value conflicts lie at the heart of the resistance to this policy innovation. Therefore, a resolution of the key value conflicts identified here—in a manner that facilitates alignment among (values in) problem framing, policy design, technology diffusion, and political decision making—will be necessary for moving the policy process forward. While the subsequent discussion paper by the union government is indicative of this realization, it is a delayed response at addressing the opposition to the policy proposal. Further, rather than enabling alignment in problem, policy, technology, and politics, it aims to minimize the role of the states in the initial implementation of the policy to circumvent the ensuing value conflicts. To promote the policy innovation, a policy design that incorporates multiple public values—both procedural and substantive—is more likely to bear fruit. Also, an ex-ante approach to the assessment of value change and value conflict—perhaps using the framework presented here—can inform future policy making and help accelerate the Indian energy transition.
